# Anti-NMDAR encephalitis with GFAPα IgG: a case report

**DOI:** 10.1186/s12883-022-02961-2

**Published:** 2022-11-12

**Authors:** Peng Bai, Bin Liu, Min Li, Mei Chun, Xiangling Ma, Jin Zhen

**Affiliations:** 1 Department of Neurology, Inner Mongolia People’s Hospital, No.20 of Zhaowuda Road, Hohhot, 010017 Inner Mongolia People’s Republic of China; 2 The Graduate School, BaoTou Medical College, No. 31 of Jianshe Road, Baotou, Inner Mongolia People’s Republic of China

**Keywords:** Anti-N-methyl-D-aspartate receptor encephalitis, Glial fibrillary acidic protein, Case study, Autoimmune encephalitis, Central nervous system, Cerebrospinal fluid

## Abstract

**Background:**

Anti-N-methyl-D-aspartate receptor (NMDAR) encephalitis is an inflammatory disease of the central nervous system (CNS) in which antibodies within the serum and cerebrospinal fluid (CSF) target NMDA receptors. Glial fibrillary acidic protein (GFAP) astrocytopathy is an autoimmune disease affecting the central nervous system (CNS). Meningoencephalitis can affect any anatomical region rostrocaudally, from the optic nerve to the spinal cord. The clinical implications of NMDAR antibodies overlapping with other antibodies against glial or neuronal cell surface proteins have not been investigated.

**Case presentation:**

A 35-year-old male presented with headaches along with amnesia, slurred and awkward speech, psychiatric symptoms, cognitive decline, and insomnia. His medical history revealed ankylosing spondylitis for six months. Ancillary findings included CSF pleocytosis and elevated protein levels. T2-weighted fluid attenuation inversion recovery was used to image high-intensity lesions of the bilateral paraventricular, radiate corona, semioval centre, and right subcortical regions. The CSF was positive for NMDAR and GFAP antibodies through transfected cell-based assays. A diagnosis of anti-GFAP encephalitis was made, although the prominent clinical features were of anti-NMDAR encephalitis.

**Conclusions:**

Herein, we describe a case of anti-NMDAR encephalitis with overlapping symptoms of GFAP antibody positivity. Patients with unusual symptoms of anti-NMDAR encephalitis should also be tested for anti-GFAP antibodies. However, because this was a single case study, caution should be exercised when interpreting the observations. Since the patient was diagnosed with autoimmune encephalitis, intravenous methylprednisolone was administered, which yielded a positive outcome.

## Background

In 2007, Dalmau et al. [1] described anti-N-methyl-D-aspartate receptor (NMDAR) encephalitis as an inflammatory condition of the central nervous system (CNS). Significant symptoms include cognitive dysfunction, speech dysfunction, seizures, abnormal behaviour, movement disorders, dyskinesia, decreased consciousness, central hypoventilation, and autonomic dysfunction [2]. Glial fibrillary acidic protein (GFAP) astrocytopathy affects the central nervous system (CNS). Although meningoencephalitis is common, any anatomical region can be rostrocaudally affected from the optic nerve to the spinal cord. GFAP is diagnosed through the detection and confirmation of immunoglobulin G (IgG) reacting with intermediate astrocyte filaments in the cerebrospinal fluid (CSF) [3, 4]. However, knowledge of disease pathogenesis and clinical outcomes is limited. There is evidence of some patients harbouring several antibodies against two or more cell-surface antigens [5–9]. However, only a few cases have been reported, and the clinical implications of NMDAR antibodies overlapping with other antibodies against glial or neuronal cell surface proteins have not been investigated [6]. Herein, we report the case of a 35-year-old male with anti-NMDAR encephalitis and autoimmune GFAP astrocytopathy, with a medical history of ankylosing spondylitis for half a year. The patient’s condition improved after treatment with intravenous (IV) methylprednisolone.

## Case presentation

A 35-year-old male had experienced headaches for one month and was subsequently admitted to our hospital department. He reported having a fever (body temperature of 38.5 °C) one month prior to presentation. His headaches began four days later, located at the forehead and bilateral temporal regions and occurring several times a day, each lasting approximately three hours. After 20 days, he began to develop amnesia, followed by mild slurring in his speech, slow and unclear speech, psychiatric symptoms (low spirit, bad mood, suicidal behaviour, and paranoia prior to hospitalisation), cognitive decline, sleep disorders usually following a temporal pattern characterised by a severe reduction in sleep duration at disease onset, and urinary incontinence. Physical examination revealed grade IV muscular strength on the right side of the body, dysarthria, a positive Kernig sign, and neck resistance. The patient’s medical history included ankylosing spondylitis for six months. He had been prescribed methylprednisolone (16 mg/day), iguratimod (40 mg/day), and sulfasalazine (2 g/day) orally. There was no history of smoking or alcohol consumption, no additional family history, and no hereditary conditions.

**Fig. 1:**
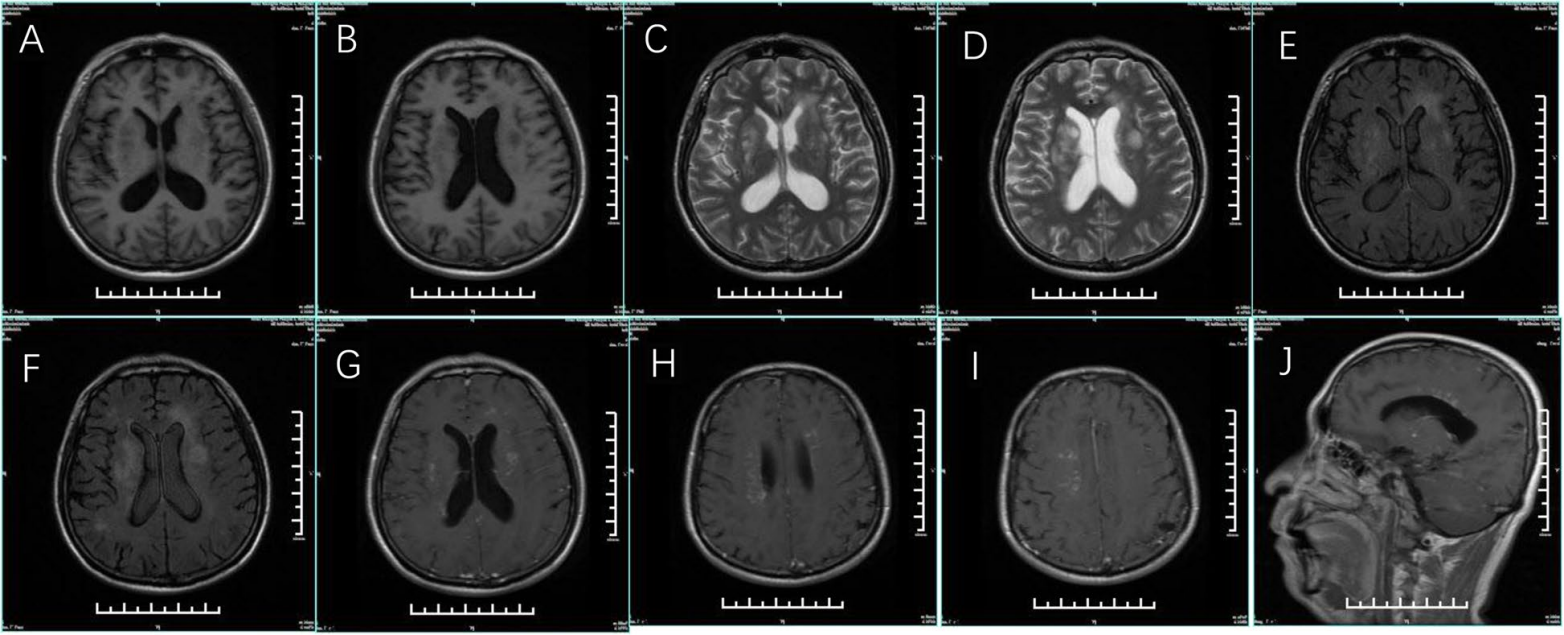
Magnetic resonance imaging of the patient. Magnetic resonance imaging of the patient. T1 weighted image revealed a low signal in the lesion area (**a**-**b**). T2-weighted fluid-attenuated inversion recovery (FLAIR) showed elevated signals within bilateral paraventricular, radiate corona, semioval center, and right subcortex (**c**-**f**), contrast-enhanced scans showed patchy and linear perivascular radial gadolinium enhancement in the above areas (**g**-**j**)

**Fig. 2:**
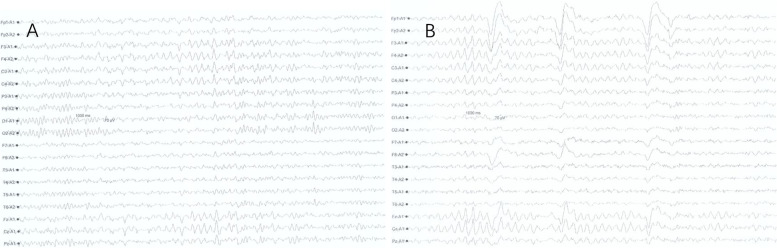
Electroencephalography of the patient. Electroencephalography (**a**-**b**) showed that low to medium wave amplitude 10-11 Hz alpha waves and alpha rhythm were seen in both occipital leads when the eyes were awake and closed, with poor amplitude and rhythm modulation and basic symmetry bilaterally. When awake and quiet, slightly more medium-wave amplitude 4-7 Hz theta waves were seen in bilateral leads, with significant anterior head, mixed with a small amount of low-wave amplitude 14-25 Hz beta waves, with basic symmetry bilaterally

**Fig. 3:**
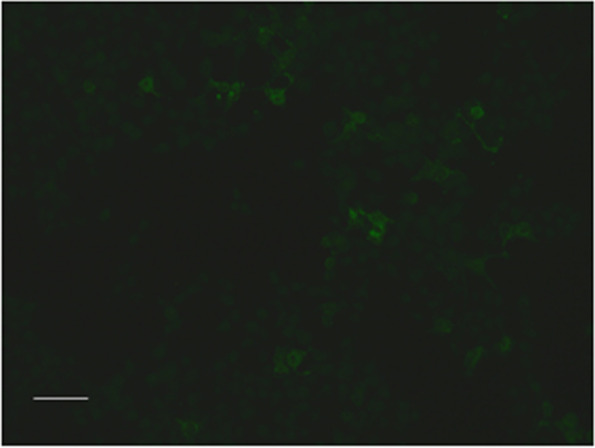
NMDAR antibodies in CSF. Anti-N-methyl-D-aspartate receptor (NMDAR) antibodies in CSF validated by a cell-based indirect immunofluorescence test (1:10)

**Fig. 4:**
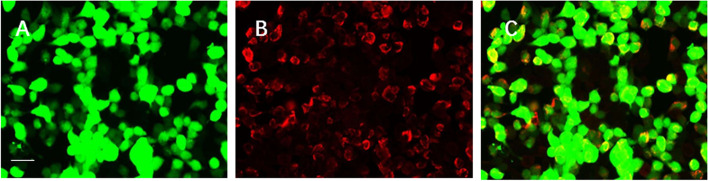
GFAPα-IgG in CSF. GFAPα-IgG test results by GFAP-transfected HEK293 cell-based immunofluorescence assay. **A** HEK293 cells expressing green fluorescent protein (GFP)-tagged GFAP (green). **B** HEK293 cells immunostained with human IgG (red if positive): **C** Merged images (yellow). Images were acquired using a Leica DM IL LED fluorescence microscope. Scale bar = 50 um

Brain magnetic resonance imaging (MRI) revealed bilateral paraventricular, corona radiata, semioval centre, and right subcortex fluid-attenuated inversion recovery (FLAIR) hyperintensities. Contrast-enhanced scans also showed patchy and linear perivascular radial gadolinium enhancement in these areas (Fig. 1). No abnormal findings were detected on nuclear MRI scans of the cervical spinal cord. While urinary incontinence, one of the patient’s symptoms, may be associated with lumbar spinal cord disease, a nuclear MRI of the lumbar spinal cord was not performed. Electroencephalography showed low to medium amplitude alpha waves of 10–11 Hz, and alpha rhythms were observed in both occipital leads when the eyes were open and closed, with poor amplitude and rhythm modulation and basic symmetry bilaterally. When awake and quiet, slightly more medium-wave amplitude 4–7 Hz theta waves were seen in bilateral leads, with significant anterior head, mixed with few low-wave amplitude 14–25 Hz beta waves and basic symmetry bilaterally (Fig. 2). The CSF pressure was normal, with leucocytosis of 94 × 106 /L (reference range: < 8 × 106 /L, proportion of neutrophils 22%), elevated protein (0.85 g/L), and normal glucose (2.95 mmol/L). Due to the patient’s psychiatric symptoms, herpes simplex virus (HSV) encephalitis was also considered. However, a CSF polymerase chain reaction (PCR) assay was negative for HSV deoxyribonucleic acid (DNA). Other viral PCRs were performed, including those for varicellovirus, cytomegalovirus, roseolovirus, lymphocryptovirus, and rhadinovirus; the results were all negative. While the patient’s CSF was positive for oligoclonal bands (OCB), his serum was negative for the same. The pattern of OCB was 2. Macroeconomic DNA detection of pathogenic microorganisms in the CSF were negative. Using a cell-based indirect immunofluorescence test (Fig. 3), anti-NMDAR antibodies were detected in the CSF (1:10). The microscope used is EUROStar III Plus fluorescence microscope. The camera model for screen imaging is LU375C. The filter system is FITC. The magnification of the microscope eyepiece is 10 times, and the objective lens is 20 times. Acquisition Software is EUROLABOFFICE. The resolution of the camera is 2048 × 1536 pixels. The resolution of the acquired image is 96 dpi. The titre changed to 1:32 when the test was repeated after eight days. Unfortunately, the patient had the test was performed at another hospital and did not provide original pictures. The serum was consistently negative for anti-NMDAR antibodies. Because contrast-enhanced scans showed patchy and linear perivascular gadolinium enhancement in the areas shown in Fig. 1, a combined GFAP antibody was considered a possibility. Cell-based assays detected anti-GFAP antibodies in the CSF (1:32) (Fig. 4), leading to the diagnosis of autoimmune encephalitis. Anti-GFAP antibodies in the CSF were validated using cell-based assays. The CSF was positive for GFAP-IgG (a-c, 1:32). Green color indicates successful transfection of a GFP-tagged plasmid encoding GFAP with a green fluorescent tag, and red represents antibody signals. Specimens were incubated with an Alexa Fluor 546 secondary antibody against human IgG (1:1000; Thermo Scientific) for 1 h at room temperature with red fluorescence. GFAP-IgG was tested by a cell-based assay (CBA). HEK293 cells were co-transfected with full-length human GFAP and pcDNA3.1-EGFP. Thirty-six hours after transfection, the HEK293T cells were fixed with 4% paraformaldehyde for 20 min and permeabilized with 0.1% Triton X-100 in phosphate-buffered saline (PBS) for 20 min. Cells were incubated with the patient's CSF for 2 h and then immunolabeled with an Alexa Fluor 546 secondary antibody against human IgG (1:1000; Thermo Scientific) for 1 h at room temperature. First, use the green light channel to observe cell transfection. If the plasmid transfection is successful, the cell can be observed to have green fluorescence. Then observe with the red channel, if the cell membrane of the successfully transfected cells in the sample well has a more obvious red fluorescence, the sample is positive for the antibody; if the cell membrane of the successfully transfected cells in the sample well does not have a more obvious red fluorescence or the cells that are not successfully transfected have red fluorescence, the sample is negative. The results can be further confirmed by overlapping the green and red channels. Two independent masked assessors classified each sample as positive or negative based on immunofluorescence intensity in direct comparison with non-transfected cells and control samples. Once confirmed, the positive specimens were then serially diluted from 1:10 to 1:1000 to determine the titers. Antibody titer is the highest dilution at which a specimen is tested for a positive reaction after a series of dilutions. The detection of GFAP antibody, cerebrospinal fluid routine non-dilution operation, and then for the positive samples, is the sampling end point dilution (end-point dilution) to determine the final titer. According to the fluorescence intensity of the test, the positive sample is further diluted at 1:10,1:100, and 1:1000 and then detected by the CBA method. Suppose the sample does not detect a positive signal when diluted at 1:100, and a positive signal is detected when diluted at 1:10. In that case, the titer is determined to be 1:10 or 1:32 (the specific situation is judged by the fluorescence intensity that can be seen when diluted at 1:10. If the fluorescence intensity is weak at 1:10 dilution detection, the titer is determined to be 1:10, and if the signal is strong at 1:10 dilution detection, the titer is determined to be 1:32). Images were acquired using a Leica DM IL LED fluorescence microscope. The filter system is L5 ET,s. The magnification of the microscope eyepiece is 10 times, and the objective lens is 20 times. The resolution of the camera is 3072 × 2048 pixels. The resolution of the acquired image is 96 dpi. However, the serum was negative for anti-GFAP antibodies. Other autoimmune antibodies, including anti-α-amino-3-hydroxy-5-methyl-4-isoxazole propionic acid 1, anti-α-amino-3-hydroxy-5-methyl-4-isoxazole propionic acid 2, anti-gamma-aminobutyric acid β, leucine-rich glioma-inactivated 1, anti-contactin-associated protein-like 2, and anti-glutamic acid decarboxylase 65-kilodalton isoform, were absent in both the CSF and serum. Other abnormal serum indicators were increased anticardiolipin antibodies, antibodies against β2-glycoprotein, human leukocyte antigen-B27, and anti-polymyositis/scleroderma (PM/Scl). A chest computed tomography scan was negative for malignancy.

**Fig. 5:**
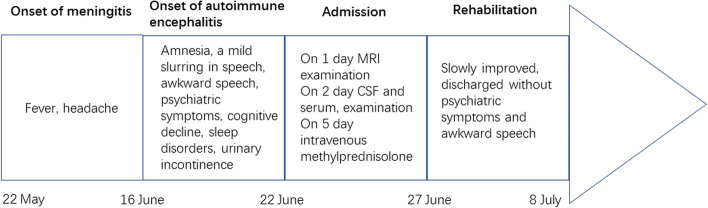
Timeline of clinical events, diagnostic-therapeutic approach, and clinical outcome

Based on the diagnosis of autoimmune encephalitis, the patient intravenously (IV) administered methylprednisolone and acyclovir; the antiviral drug acyclovir, used to treat HSV infections, was administered even though CSF PCR analysis was negative for HSV DNA. The patient was also treated with venous antibiotics, B vitamins, and other supportive measures. His psychiatric symptoms were in remission after ten days of IV methylprednisolone treatment. No serious adverse events occurred during this period, and the patient was subsequently discharged. Prednisolone acetate and mycophenolate mofetil were prescribed as maintenance therapies. This treatment regimen was well-tolerated with no side effects. Further cancer screening is planned for the patient’s follow-up appointments. Figure 5 depicts the patient's clinical milestones. We used PhotoshopCS3 to adjust the resolution of all the figures.

## Discussion and conclusions

This report describes a case of anti-NMDAR encephalitis associated with overlapping symptoms and positive antibodies against GFAP. The patient was a 35-year-old male with symptoms of headaches, memory barrier, incoherent speech, psychiatric symptoms, urination, and sleep disorder. T2-weighted FLAIR signal hyperintensities were observed for the cerebral cortex, frontonasal and basal ganglia, and the CSF was positive for anti-NMDAR antibodies. In this case, anti-NMDAR encephalitis was confirmed according to recently established criteria [2].

Tests for GFAP-IgG in the CSF and tissue are biomarkers of autoimmune GFAP astrocytopathy. The detection of autoantibodies in CSF against GFAPα is the most clinically sensitive and specific diagnostic biomarker for this disease [8, 10]. Several studies [3, 8, 10, 11] have described the clinical and immunological characteristics of autoimmune GFAP astrocytopathy. It is rare to have isolated myelitis when GFAP-IgG is present in the CSF. Meningoencephalitis is the most common phenotype, but isolated myelitis does occur occasionally. The presence of flu-like symptoms is also common (40–66% of cases). In addition to encephalopathy, antiepileptic drug-resistant seizures, psychiatric symptoms, tremors, and meningeal symptoms, the condition is most commonly accompanied by encephalopathic features [4, 8]. The disease is reactive to unique corticosteroids, indicating encephalitis or its limited form, and is accompanied by lymphocytic and multi-cytoplasmic features on MRI scans, with reduced flow in the cerebral linear vessel [8, 10, 12]. Abnormal hyperintensities are most common in the basal ganglia, followed by the thalamus [10]. Coexisting GFAP-IgG is sometimes accompanied by NMDA-R-IgG [8, 9]. In some patients, NMDAR autoimmunity increases the possibility of a primary inflammatory event initiated by an additional plasma membrane protein-directed IgG, disrupting astrocytic function and leading to GFAP autoimmunity as a secondary phenomenon. The reason for GFAP antibodies being present in anti-NMDAR encephalitis patients is unclear. While NMDAR antibodies account for the clinical symptoms of patients, GFAP is an accompanying antibody that only causes imaging changes [4]. The autopsy results in one patient positive for GFAP antibodies were nonspecific and showed no astrocyte involvement [13]. Currently, there are no uniform diagnostic criteria for GFAP astrocytopathy [3, 14]. Patients with anti-NMDAR encephalitis combined with GFAP antibodies may differ from those with anti-NMDAR encephalitis alone in terms of clinical presentation, imaging, and treatment response. Further immunohistochemical studies may provide insights into the disease mechanisms [8].

Recently, IgG antibodies directed against neuronal surfaces or glial antigens have helped diagnose autoimmune encephalitis [6, 15]. In general, the type of antibody determines the symptoms, syndrome specificity, frequency, and type of tumor association [6]. Clinical diagnosis of immune-related disorders is based on these symptoms. It is possible, however, to overlook an immune response or attribute clinical features to atypical manifestations of a single immune response. As a result, one of the associated diseases or an associated underlying tumor may be initially undiagnosed.

Recently, a clinical overlap between myelin oligodendrocyte glycoprotein (MOG) encephalomyelitis associated with anti-NMDAR antibodies (NMDARE) and MOG antibody disease associated with anti-NMDAR antibodies (MNOS) was found. It is common for both MOG and NMDAR antibodies to be positive in these diseases. MNOS can be characterised by either MOG encephalomyelitis or NMDARE; at times, it is also characterised by both clinical manifestations. However, an overlapping syndrome is associated with milder clinical manifestations than NMDARE alone [9]. Therefore, medical professionals should monitor patients with anti-NMDAR encephalitis for the development of optic neuritis, myelitis, or other demyelinating diseases that are not consistent with autoimmune encephalitis. Serological evaluation of antibodies associated with demyelinating diseases is important. There the possibility of coexisting immune-mediated encephalitis should be considered when atypical symptoms occur simultaneously with MOG encephalomyelitis or other demyelinating disorders [16].

An unexpected finding in this case was that the patient had a history of ankylosing spondylitis, since the relationship between ankylosing spondylitis and autoimmune encephalitis remains unclear. A highlight of this case was that the patient suffered from ankylosing spondylitis with an inflammatory CNS disease. A medical history of ankylosing spondylitis could of particular interest in patients with similar symptoms.

Patients with anti-NMDAR encephalitis should be evaluated for GFAP antibodies, despite the underlying mechanism remaining unclear. Because of the facts of a single case study, caution should be exercised when explaining the observational results. The exact relationship between these two antibodies must be determined through further studies.

## Data Availability

All data analyzed during this study are included in this article.

## Abbreviations

NMDAR: N-methyl-D-aspartate receptor
CNS: Central nervous system
CSF: Cerebrospinal fluid
GFAP: Glial fibrillary acidic protein
IgG: Immunoglobulin G
IV: Intravenous
MRI: Magnetic resonance imaging
FLAIR: Fluid-Attenuated Inversion Recovery
HSV: Herpes simplex virus
PCR: Polymerase chain reaction
DNA: Deoxyribonucleic acid
PM/Scl: Polymyositis/scleroderma
CT: Computed tomography
IgM: Immunoglobulin M
EEG: Electroencephalography
OCB: Oligoclonal bands
Ab: Antibody
NMDARE: Anti-NMDAR encephalitis
MNOS: MOG antibody disease and NMDARE overlapping syndrome
AMPA1: α-Amino-3-hydroxy-5-methyl-4-isoxazole propionic acid 1
AMPA2: α-Amino-3-hydroxy-5-methyl-4-isoxazole propionic acid 2
LGI1: Leucine-rich glioma-inactivated 1
CASPR2: Contactin-associated protein-like 2
GAD65: Glutamic acid decarboxylase 65-kilodalton isoform
